# Depression as a Neuroendocrine Disorder: Emerging Neuropsychopharmacological Approaches beyond Monoamines

**DOI:** 10.1155/2019/7943481

**Published:** 2019-01-03

**Authors:** Mervin Chávez-Castillo, Victoria Núñez, Manuel Nava, Ángel Ortega, Milagros Rojas, Valmore Bermúdez, Joselyn Rojas-Quintero

**Affiliations:** ^1^Endocrine and Metabolic Diseases Research Center, School of Medicine, University of Zulia, Maracaibo, Venezuela; ^2^Psychiatric Hospital of Maracaibo, Maracaibo, Venezuela; ^3^Universidad Simón Bolívar, Departamento de Ciencias Sociales y Humanas, Cúcuta, Colombia; ^4^Division of Pulmonary and Critical Care Medicine, Brigham and Women's Hospital and Harvard Medical School, Boston, MA 02215, USA

## Abstract

Depression is currently recognized as a crucial problem in everyday clinical practice, in light of ever-increasing rates of prevalence, as well as disability, morbidity, and mortality related to this disorder. Currently available antidepressant drugs are notoriously problematic, with suboptimal remission rates and troubling side-effect profiles. Their mechanisms of action focus on the monoamine hypothesis for depression, which centers on the disruption of serotonergic, noradrenergic, and dopaminergic neurotransmission in the brain. Nevertheless, views on the pathophysiology of depression have evolved notably, and the comprehension of depression as a complex neuroendocrine disorder with important systemic implications has sparked interest in a myriad of novel neuropsychopharmacological approaches. Innovative pharmacological targets beyond monoamines include glutamatergic and GABAergic neurotransmission, brain-derived neurotrophic factor, various endocrine axes, as well as several neurosteroids, neuropeptides, opioids, endocannabinoids and endovanilloids. This review summarizes current knowledge on these pharmacological targets and their potential utility in the clinical management of depression.

## 1. Introduction

Depression is one of the most frequent mental disorders in everyday clinical practice and is currently regarded as the leading cause of disability worldwide [1]. In addition to the profoundly debilitating condition of this disorder, major depressive disorder (MDD) entails an increased risk of medical comorbidities [2] and very high direct and indirect financial costs [3]; profiling this disorder as an important problem for public health.

In spite of this outlook, pharmacotherapy alternatives for MDD remain insufficient: Currently available antidepressant drugs (AD) have only been shown to achieve remission rates around 56% after four successive treatment stages [4]. Moreover, a majority of the available AD at present display problematic side-effect profiles and a delayed onset of action, further complicating the management of this disorder [5]. The development of newer, more effective, and tolerable agents is a pressing matter in neuropsychopharmacology, yet relatively few new drugs have been approved for MDD in recent decades [6].

Both the limited effectivity of existing AD and the scarcity of novel options may stem from a once revolutionary, yet—in retrospect—excessive and misguided focus on the monoamine hypothesis for the pathophysiology of depression, which centers on defective neurotransmission of serotonin (5-hydroxytriptamine, 5HT), noradrenaline (NA), and dopamine (DA) in the brain [7]. Indeed, the serendipitous discovery of tricyclic AD drove the “reverse engineering” of this hypothesis, which in turn has guided much of the development of all AD throughout history [8]. Nevertheless, the monoamine hypothesis has been heavily contested regarding its validity and the relative importance of its components [9, 10]. At present, advances in molecular psychiatry have reframed neuronal monoamine dysregulation to be the end state of a complex interplay among pathophysiologic pathways involving several nonmonoamine neurotransmitters, as well as several endocrine-metabolic components [11].

This more holistic understanding of the pathophysiology of MDD has allowed for the design and investigation of novel and promising AD candidates, with activity outside the monoamine dysregulation end state, thus providing provocative windows for intervention [12]. As preclinical and clinical studies progress at various rates for these molecules, this review aims to summarize current views on the neurobiology of depression, with an emphasis on emerging pharmacological targets beyond monoamine neurotransmission.

## 2. Expanding Views on the Neurobiology of Depression

**Figure 1:**
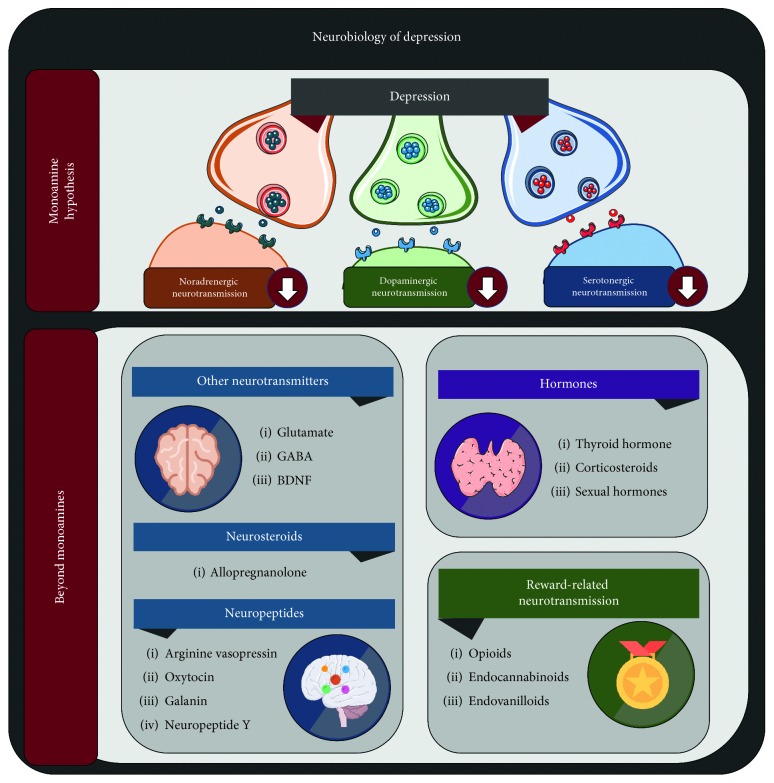
Expanding views on the neurobiology of depression. GABA: *γ*-aminobutyric acid; BDNF: brain-derived neurotrophic factor. Current neuropsychopharmacological approaches to depression are centered on the monoamine hypothesis. Nevertheless, the imperfect results obtained in clinical practice with currently available antidepressant drugs have propelled the discovery of various potential pharmacological targets beyond noradrenaline, dopamine, and serotonin.

The understanding of depression as a clinical entity has evolved radically, from the early descriptions of Hippocrates' *melancholia* and other primitive pre-Kraepelinian conceptualizations to the rich variety of descriptions derived from various psychological currents during the 20th century, to the revolutionizing contributions of psychopharmacology and neurobiology in more recent history [13]. Research advances in the latter fields have particularly propelled medical models for depression and mental disorders in general, marking a transition in the understanding of these diagnoses from rather intangible, elusive concepts, to more concrete biological terms, especially centering on the monoamine hypothesis [14]. However, novel approaches exceed and intertwine with this central dysfunction in monoamine neurotransmission, by involving other neural, endocrine and metabolic pathophysiologic components (Figure 1). Firstly, neurotransmitters beyond the three classic monoamines will be discussed in the following paragraphs.

### 2.1. Glutamate: A Versatile Regulator

Glutamate (Glu) is the major excitatory neurotransmitter in the mammalian brain [15]. In normal circumstances, Glu plays a prominent role in synaptic plasticity, learning, and memory. However, in pathological conditions, it is known to be a potent trigger for rapid or delayed neurotoxicity [16]. With emerging findings of antidepressant effects for glutamatergic drugs, speculation has arisen about the role of Glu in the pathophysiology of mood disorders [17]. In particular, depressed patients appear to have increased basal glutamatergic activity. As a result, preclinical and clinical studies with drugs directly targeting glutamatergic neurotransmission present new and provocative opportunities for antidepressant treatment [18].

Glu receptors are divided into two major families: ionotropic and metabotropic glutamate receptors (mGluRs). The ionotropic group includes NMDA, AMPA, and kainate receptors [19]. In resting conditions, NMDA receptors are blocked by magnesium until membrane depolarization, when the combined binding of two Glu molecules and two molecules of glycine or D-serine allows the influx of calcium, serving as a functional marker of converging excitatory input and ultimately producing excitation over longer periods of time [20]. AMPA receptors mediate the fast rapidly desensitizing excitations at most synapses and are responsible for the initial reaction to Glu in the synapse. Their activation permits the influx of sodium resulting in the depolarization of the neuronal membrane. Like AMPA receptors, kainate receptors are associated with voltage-dependent channels that primarily allow for the influx of sodium ions that mediate fast excitatory neurotransmission, but they appear to be less widespread and have a distinct distribution [21]. On the other hand, the metabotropic family consists of group I receptors (mGluR1 and mGluR5), which potentiate both presynaptic glutamate release and postsynaptic NMDA currents, and group II (mGluR2 and mGluR3), and group III receptors (mGluR4, mGluR6, mGluR7, and mGluR8), which tend to suppress glutamate function [19, 22].

To date, evidence has emerged indicating that NMDA receptor antagonists, group I metabotropic glutamate receptor (mGluR1 and mGluR5) antagonists, and positive modulators of AMPA receptors have antidepressant-like activity in a variety of preclinical models [23]. Historically, the preclinical antidepressant-like effects of the NMDA receptor antagonists AP-7 and MK-801 first suggested Glu signaling to be a potential therapeutic approach [24].

This research led to the experimental use of ketamine a noncompetitive NMDA receptor antagonist, which was profiled as a reasonable candidate for psychiatry given the previous years of safe and well-tolerated use in the fields of anesthesia and neurology [25]. Ketamine has been shown to induce rapid antidepressant effects within 24 hours of use at subanesthetic doses, lasting for at least several days after a single infusion in various blind pilot clinical trials, which has led to the coining of the term “rapid-acting antidepressants” (RAA) [26–28]. This effect has been reported to begin after the initial psychotomimetic, dissociative, and euphoric effects have subsided, suggesting that the antidepressant effects are not just a result of acute elevated mood [29]. Nonintravenous ketamine preparations, such as oral and intramuscular forms, have also shown antidepressant efficacy [30, 31]. Intranasal ketamine appears most promising, owing to its high penetrance into the central nervous system and ease of administration [32]. This rapid action has prompted speculation posting glutamatergic neurotransmission as the key pharmacological target to bypass the delay in the onset of action of classic monoaminergic drugs. Nonetheless, concerns remain surrounding the use of ketamine as an antidepressant due to its pharmacological similarity to the potent psychotomimetic drug, phencyclidine (PCP), and its abuse liability as a hallucinogenic club drug [33].

**Figure 2:**
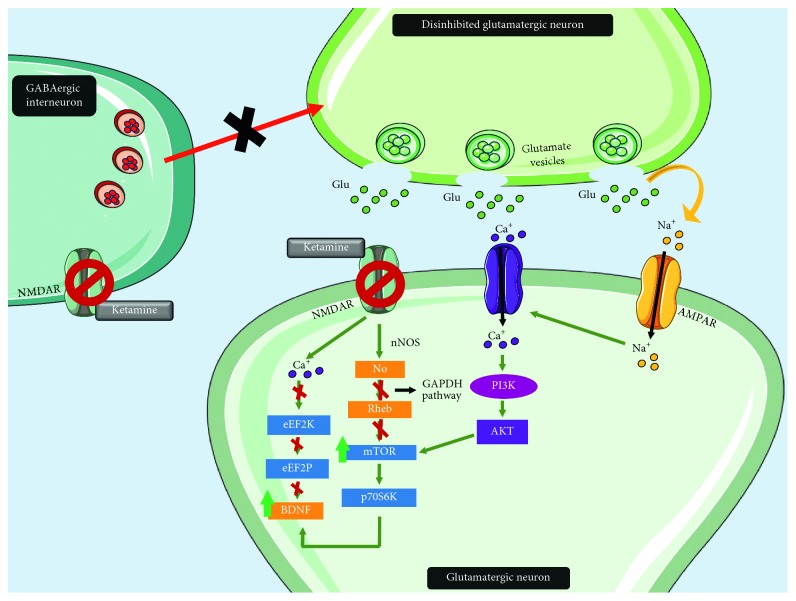
Effects of ketamine on the glutamatergic synapse. Ketamine acts as an NMDA antagonist on GABAergic interneurons, as well as on postsynaptic glutamatergic neurons. Antagonism in the former results in disinhibition of presynaptic glutamatergic neurons, thus favoring activation of AMPAR in postsynaptic glutamatergic neurons. This, along with activation of voltage-dependent calcium channels, results in activation of the PI3K pathway which leads to increased mTOR activity. Furthermore, antagonism of NMDAR leads to inhibition of the nitric oxide pathway, which in turn leads to Rheb stabilization and mTOR pathway potentiation. mTOR increases p70s6K activity, which promotes BDNF signaling. BDNF activity is also favored by the inactivation of eEf2K secondary to NMDAR antagonism.

Ketamine displays an interesting pharmacologic profile, as it is a *µ*-opioid receptor agonist with superior affinity for NMDA receptors [33]. Ketamine antagonizes NMDA receptors on GABAergic interneurons and on postsynaptic neurons, resulting in disinhibition of cortical glutamatergic neurons through the former [34] and increased synthesis of intracellular growth factors through the latter [22]. In addition, ketamine promotes inhibition of spontaneous NMDA receptor-mediated excitatory postsynaptic currents. In turn, this leads to suppression of elongation factor 2 kinase activity (eEF2K), permitting a rapid increase in the translation of brain-derived neurotrophic factor (BDNF), an important mediator for neuroplasticity and neuroprotection [35]. Ketamine has also been noted to inhibit signaling by nitric oxide, which allows for the stabilization of nitrergic Rheb, a small G protein that enhances intracellular signaling [36]. Indeed, ketamine seems to activate many intracellular cascades, including the mammalian target of rapamycin (mTOR) pathway, which has been observed to lead to an increased number and function of new synapses in the prefrontal cortex (PFC) of rats, reverting the synaptic deficits that result from exposure to stress [22, 37]. Furthermore, ketamine appears to increase p70 s6 kinase (p70s6K) and 4E-binding protein phosphorylation, both involved in the modulation of synaptogenesis (Figure 2) [37].

The promising results with ketamine have ignited considerable research on other rapid-acting antidepressant molecules [38]. Esketamine and arketamine—the *(S)* and *(R)* isomers of ketamine—have also been evaluated in both preclinical and clinical studies. In particular, in animal models, arketamine appears to induce more potent and longer-lasting antidepressant effects than esketamine without psychotomimetic effects [39]. Nevertheless, clinical research on arketamine is scarce to date, while esketamine appears to be effective for the acute improvement of depressive symptoms, yet less potent than ketamine, and with a similar side-effect profile [40]. Finally, *(S)*-norketamine, another ketamine derivate, has been reported to induce rapid and long-lasting antidepressant effects in rodent models with lower potency than esketamine, yet without psychotomimetic and other detrimental biochemical and neurophysiologic effects [41]. Indeed, with further investigation, ketamine-related molecules could strike a balance between clinical effectivity and tolerability in the relatively near future.

Repastinel is another RAA unrelated to ketamine, a tetrapeptide derived from the light chain of the B6B21 monoclonal antibody which acts as a NMDA receptor modulator and glycine-site partial agonist [41]. In animal models, repastinel appears to promote long-term potentiation of electrophysiological activity in the hippocampus and PFC by enhancing NMDA receptor-dependent signaling [42]. Although clinical research on repastinel is only in its early stages, repastinel appears to induce rapid and sustained antidepressant effects and be well-tolerated with no psychotomimetic effects [43].

In contrast with these rather selective glutamatergic agents, scopolamine, a nonselective muscarinic receptor antagonist, also displays Glu-modulating activity, which may correlate with antidepressant effects [44]. Animal models show that, like ketamine, scopolamine is a RAA, increasing glutamatergic neurotransmission and activation of mTORC1 signaling in the PFC [22, 45]. This enhancement is thought to occur via blockade of muscarinic receptors located on GABAergic interneurons in the medial PFC, similar to the initial cellular target underlying the actions of ketamine [46]. Several clinical studies have reported rapid antidepressant effects following intravenous infusion of low doses of scopolamine, and repeated doses (3 doses over 5–7 days) produced long-lasting improvements in mood for up to two weeks [47, 48]. In this scenario, scopolamine has shown more efficacy in treatment-naïve patients, although treatment-resistant patients still show significant reductions in depressive symptoms [49].

Because of the current limitations of ketamine, its derivatives, and other emerging RAA, such as psilocybin, which is unrelated to ketamine and its associated mechanisms of action, yet also presents the clinical dilemma of rapid clinical effectivity vs tolerability and psychotomimetic effects [50–52], research has been conducted to seek more tolerable NMDA receptor antagonists that could replicate its antidepressant effect with less adverse effects. Memantine, a NMDA receptor antagonist currently approved for the management of Alzheimer's disease, has been studied in mood disorders. Preclinical reports in rodent models of depression have noted antidepressant-like effects [53]. However, currently available clinical placebo-controlled trials have failed to demonstrate efficacy on depressive symptoms in humans [54, 55].

Riluzole, an FDA-approved medication for the treatment of amyotrophic lateral sclerosis, has also been used in some ketamine extension studies [56, 57]. It is a Glu receptor modulator [58] with apparent efficacy as both monotherapy [52] and adjunctive therapy in patients with treatment-resistant major depressive disorder (MDD) [59]. Research has found this antidepressant effect to be especially notable at 4 weeks in treatment-resistant populations, although placebo-controlled studies tend to deny any significant differences [60]. Therefore, further investigation is required to better characterize the clinical efficacy of riluzole in this context. Pharmacodynamic studies suggest riluzole is not an open-channel antagonist of the NMDA receptor and rather acts by enhancing the surface expression of AMPA receptor subunits in cultured hippocampal neurons, resembling the activity of lamotrigine in this aspect [61]. Riluzole has also been reported to stimulate BDNF expression [62] and accelerate Glu clearance from the synaptic cleft by facilitating Glu reuptake by astrocytes [63]. Both of these effects have been associated with antidepressant-like effects in preclinical rodent models [64].

There are currently several other Glu-modulating molecules under early experimental study for depression. Ro(25)-6981, an NMDA receptor antagonist with selectivity for the NR2B subunit has shown antidepressant-like effects, possibly mediated by increased expression of postsynaptic cascades such as the mTOR pathway [21]. A similar candidate, MK-801 (dizocilpine), is a high-affinity, noncompetitive antagonist of NMDA receptors with comparatively inconsistent antidepressant effects [65]. Although both appear to have significant antidepressant effects in the short term, neither appear to be as long-acting as ketamine [21, 66].

Metabotropic Glu receptors have also been examined as potential therapeutic targets in depression [67]. LY341495, an mGlu2/3 receptor antagonist, and not LY379268, an agonist for this receptor group, has been associated with an antidepressant effect [68, 69]. This appears to involve activation of the mTORC1 pathway, in a similar fashion to MGS0039, another mGlu2/3 receptor antagonist, as well as ketamine. LY341495 also rapidly reverses anhedonia caused by chronic stress exposure in animal models, a rigorous rodent test of rapid antidepressant actions [70].

Indeed, stimulation of the mTOR cascade, as well as concurrent activation of AMPA receptors in the PFC may be essential for the antidepressant effect linked to Glu modulators [71]. The importance of AMPA receptors as mediators of antidepressant effects has been highlighted by Alt et al. [72]: Certain structural variants of AMPA receptors may be associated with modulation of AMPA receptor-mediated currents. In turn, this appears to facilitate monoaminergic neurotransmission by promoting BDNF signaling.

On the other hand, a study by Palucha-Poniewiera et al. in rats reported short-lived antidepressant effects for both MTEP, an mGlu5 receptor antagonist, and AMN802, an mGlu7 receptor agonist. Their onset of action began within 60 minutes of administration and disappeared at around 23 hours after. Results showed MTEP did not promote mTORC1 signaling; whereas AMN802 stimulated this pathway, yet failed to concurrently activate AMPA receptors [66]. Insights into this class of molecules are still in early experimental phases and further research is required.

Lastly, both zinc and magnesium have shown antidepressant activity in preclinical [73–75] and clinical studies [76–78] and may be valuable adjunctive agents in the pharmacotherapy of depression. Zinc and magnesium appear to decrease the activity of NMDA receptors, in association with increased BDNF and glycogen synthase kinase-3 (GSK-3) signaling.

### 2.2. GABA: From Anticonvulsants to Mood Stabilizers to Antidepressants?

*γ*-Aminobutyric acid (GABA) is the main inhibitory neurotransmitter on the mammalian brain, and its various receptors are widespread in the central nervous system [79]. Two general classes of GABA receptors are known: in the GABA-A family, the receptor is part of a ligand-gated ion channel complex where each isoform consists of five homologous or identical subunits surrounding a central chloride ion-selective channel gated by GABA. They are located in the postsynaptic membrane and drive immediate neuronal inhibition, while those located in extrasynaptic membranes respond to ambient GABA conferring long-term inhibition [80]. In contrast, the GABA-B family includes metabotropic G protein-coupled receptors that open or close ion channels via a second messenger system [81]. GABA-modulating agents, such as anticonvulsant drugs (ACD), are widely known to be effective in the management of depression [82]. This effect has been proposed to be due to a more favorable Glu-GABA balance driven by ACD. However, the distinct mood-regulating pattern of each ACD, as well as the absence of mood regulation by some ACD, suggests the antidepressant pharmacodynamics of GABAergic drugs to be more complex than solely a shift in the Glu-GABA equilibrium [83].

Reports have demonstrated increased GABA-B receptor binding in rodent brains after chronic administration of several antidepressant drugs [84], supporting a GABAergic hypothesis of antidepressant drug action. Nevertheless, further research has been inconsistent regarding the purported antidepressant effect of GABA-B receptor binding [85]. Currently, the proven clinical efficacy of ACD in mood disorders continues to fuel the study of GABAergic modulation in this context [86].

Virtually, all of the known molecular components of the GABA and Glu systems have been considered as potential therapeutic targets [87]. In particular, negative allosteric modulators of GABA-A receptors containing *α*5 subunits (*α*5 GABA-NAMs) have been proposed as a novel class of rapid-acting antidepressants. These produce a similar net effect to that of ketamine in the forebrain, yet with reduced side effects due to the comparatively lower expression of *α*5 subunits in this region. In a study on male mice, MRK-016, a *α*5 GABA-NAM, was associated with an antidepressant-like response, without changes in side effect-monitoring parameters, such as rota-rod performance, conditioned-place preference, and locomotion [88]. Further research is required to better characterize the usefulness and suitability of this promising molecule in humans.

### 2.3. Brain-Derived Neurotrophic Factor

Decreased hippocampal expression of BDNF is a well-recognized component of the neurobiology of depression, anxiety, and stress, and antidepressant treatment has been observed to increase levels of this messenger [89]. By activating signaling mediated by tropomyosin-related kinase receptor (TrkB), BDNF plays a central role in neurogenesis and neuronal survival and growth [90, 91]. Although BDNF was initially proposed as a therapeutic alternative for depression, clinical trials with recombinant BDNF have failed to achieve significant antidepressant efficacy [92, 93].

Recent efforts have been directed to find other ways to exploit the beneficial effects of BDNF signaling, specifically through the study of TrkB ligands [94]. In particular, the use of 7,8-dihydroxyflavone, a TrkB agonist, has shown antidepressant activity in mice models [95, 96], along with promotion of neurogenesis [97, 98]. On the other hand, ANA-12 is a selective TrkB antagonist that can inhibit its neurotrophic activity in the nucleus accumbens without compromising neuronal survival. Paradoxically, this antagonist has also been linked with antidepressant and anxiolytic activity in mice models [99, 100]. Therefore, further research is needed to clarify the pharmacology and clinical correlates of the use of TrkB ligands for depression.

## 3. Endocrine Pharmacotherapeutic Targets in Depression

The fields of psychiatry and endocrinology have long been known to be largely interrelated with various mental and endocrine disorders often displaying bidirectional relationships [101]. Depressive and anxious symptoms are common in subjects with hormonal disturbances and may represent a challenge for clinicians [102]. Although patients with depression do not always have overt or severe endocrine disease, hormonal changes are frequent in this population, with accumulating evidence supporting a pathophysiologic role in the context of depression, and by extension, endocrine disturbances may be a therapeutic target in this disorder [103].

Thyroid hormone is notoriously involved in brain development and function, and neuropsychiatric manifestations are hallmarks of thyroid disease [104]. Conversely, psychiatric disorders often feature disruptions of the hypothalamus-pituitary-thyroid axis (HPTA): Patients with depression have been found to show abnormal responses to thyroid-stimulating hormone (TSH) and thyrotropin-releasing hormone (TRH), as well as elevated TRH concentrations in cerebrospinal fluid and increased prevalence of antithyroid antibodies [105]. Available data to date indicates overt thyroid pathology is rare in subjects with depression [106]. On the contrary, subclinical thyroid pathology appears to be a significant risk factor for psychiatric disorders [105], with multiple studies demonstrating depressive symptoms to be significantly more frequent or severe in patients with subclinical hypothyroidism than in age- and sex-matched controls [107–109]. This outlook outlines the complexity of the involvement of the HPTA in the pathophysiology of depression.

Indeed, the role of the HPTA in depression may be subtler than gross thyroid disease, articulated by the ubiquity of thyroid hormone receptors in all tissues, and the susceptibility of this system to stress. Dynamic adjustments of the HPTA represent a physiological response in stress conditions, such as intense physical activity and pregnancy [110]. However, in depression, a chronic state of low-grade systemic inflammation appears to disrupt the HPTA and many other endocrine and immune axes [111]. In vitro and in vivo studies have shown T3 to stimulate microglial migration and activation, a prominent phenomenon seen in depression, schizophrenia, and autism spectrum disorders [112, 113]. In this context, T3 signaling appears to result in disruptions in the PI3K, and MAPK/ERK cascades, as well as potentiated nitric oxide-mediated microglial migration and activation [114–116]. These events could contribute to the dysfunctions in neurotrophism, neuroplasticity, and neurotransmission seen in depression [117].

Administration of thyroid hormone is a well-supported augmenting strategy in the management of refractory depression, even in euthyroid patients [118–120]. However, hormone replacement alone may be insufficient to treat depression in hypothyroid patients, and further research is needed to better characterize the profile of patients who would benefit most from this intervention [121].

Corticosteroids have also been widely recognized to play a role in the neuroendocrine disturbances found in depression. Cortisol, the final product of the hypothalamus-pituitary-adrenal axis (HPAA), is broadly regarded as the “stress hormone,” mediating a myriad of essential functions across all organ systems under acute and chronic stress conditions [122]. However, cortisol is also a notorious participant in the pathophysiology of chronic stress-related illnesses, as seen in depression [123]. Central corticotropin-releasing hormone (CRH) hyperstimulation appears to be a key perpetuating factor of chronic stress and depression, and it has been suggested that suppression of CRH activity might be the final and common step of antidepressant action that is necessary for the stable remission of MDD [124]. Two primary CRH receptor subtypes—CRHR1 and CRHR2—have been described in the central nervous system (CNS) according to their neuroanatomical expression patterns; with CRHR1 appearing to play a key role in mediating the CRH-elicited effects in depression and anxiety [125]. Specific CRHR1 haplotypes have been linked with the development of MDD [126]. Therefore, the CRHR1 gene may be considered a candidate gene for antidepressant pharmacogenetics [127].

Evidence over the years for increased CRH activity initially led to the development of CRH receptor antagonists as putative treatments for depression [123]. A prominent member of this group is R121919, which has been shown to alleviate anxiety in rats and primates, as well as reduce depression scores with good tolerability in a small study with 20 subjects diagnosed with MDD [128]. NBI-30775/R121919, another CRH1 receptor antagonist, has been reported to have a clinical profile and efficacy comparable to paroxetine [129]. In contrast, CP-316,311, a selective nonpeptide CHRH1 antagonist, has failed to show efficacy in the treatment of MDD, despite being well-tolerated [130]. NBI-34041, another CRH1 receptor antagonist, has been reported to reduce stress-related secretion of cortisol upon administration, without systemic adverse effects [131]. Thus, CRH1 receptor antagonists may be promising novel therapeutic options for depression and anxiety in the future. These advancements will need to circumvent certain currently recognized difficulties regarding the pharmaceutic formulations and pharmacokinetics of these compounds, in order to assure sufficient bioavailability and penetration of the blood-brain barrier [132].

Sexual hormones have also been implicated in the neurobiology of depression, particularly regarding their very characteristic fluctuations throughout the life cycle and their correlation with mood disorders, in both sexes [133]. Episodes of mood disorders have been found to be more prevalent during key life periods with important hormonal changes, such as puberty, menopause, and the postpartum period [133–135]. Indeed, females are subject to a wider array of fluctuations with more dramatic consequences, in consonance with a more than doubled risk of mood disorders, in comparison to men [136, 137]. This disparity highlights a potential role for gonadal hormones in the etiology of mood disorders. Estrogen receptors are thoroughly distributed in the brain [138], with these hormones participating in the organization of developing neurons, and the activation of mature neurons. Estrogens drive neurite growth and synaptogenesis, augment BDNF activity, and modulate neurotransmission of 5HT, NA, DA, Glu and Ach [139]. In particular, estrogens increase serotonergic activity by regulating 5HT metabolism and 5HT receptor expression, as well as modulating the spontaneous firing of the serotonergic neurons in the raphe nuclei [139].

In a clinical context, the role of estrogens in depression has been more thoroughly studied during the perimenopausal period. Several studies have focused on estrogen replacement therapy (ERT) as a way to alleviate depression, with interesting results. In a placebo-controlled trial on perimenopausal women by Schmidt et al. [140], ERT was linked with partial or full remission of depressive symptoms as early as 3 weeks after initiating treatment, while Klaiber et al. [141] reported similar benefits for high-dose ERT in perimenopausal women with MDD over 3 months. A larger study included 661 perimenopausal women who were divided into various groups: receiving oral estrogens and progesterone, receiving transdermal estradiol and progesterone, and a placebo group. After a 48-month follow-up, women who received oral ERT showed significant relief of depressive symptoms in comparison to the placebo group [142].

Nevertheless, a meta-analysis of 10 studies with 1,208 perimenopausal women ascertained supplementation with bioidentical estrogens to have no significant effects on depressive symptoms, even with adjunctive progestogens, despite being effective in the management of vasomotor symptoms [143]. Furthermore, the KNHANES study determined prolonged oral contraceptive use and ERT usage to be linked with a significantly higher risk of depression [144]. Therefore, the clinical use of estrogens for depression remains controversial and requires further research.

Similar to what occurs in menopause, the sudden drop in estradiol levels seen after delivery has been hypothesized to contribute prominently to postpartum depression (PPD) [138]. Moreover, fluctuations in progestogen levels may also intervene significantly: Progesterone and other progestogens have been related to negative mood states, possibly by disrupting GABAergic neurotransmission [145]. In postmenopausal women treated with progesterone and animals treated with allopregnanolone (APG), there is a bimodal association between serum APG concentration and adverse mood, resembling an inverted U-shaped curve. Moreover, in humans, the maximal effective concentration of APG for producing negative mood is within the range of physiological luteal-phase serum concentrations [146].

Clinical data on the use of estrogens or progestogens in PPD is currently scarce, although available results suggest treatment with sublingual estradiol to be linked with rapid reduction of depressive symptoms [147]. Administration of transdermal E2 has also been considered as a possible therapeutic pathway, considering that in this way, hepatic metabolism is by-passed and risk for venous thromboembolism decreases. However, recent pilot studies have been unsuccessful as of yet [148]. What has been considered a promising target in postpartum depression has been the aforementioned APG. In a randomized controlled trial, Kanes et al. researched the use of an intravenous dose of APG, finding scarce adverse events and significant improvement of the symptoms [149]. Further studies are required to elucidate the role of hormone therapy in PPD, as this entity continues to be recognized as remarkably difficult to treat.

Finally, androgens of both adrenal and gonadal origin can cross the blood-brain barrier, with multiple effects on the brain, and various androgens, including testosterone, can be synthesized *de novo* in the brain [150, 151]. Androgens act as allosteric modulators of GABA-A receptors, increasing the duration and frequency of the opening of their associated chloride channel [152], modulating various neurotransmitter systems and neuronal excitability, with important implications in the neurobiology of mood disorders [152–155]. This profile appears to have significant clinical correlations: Testosterone levels decline progressively with age, in association with symptoms intriguingly similar to those seen in depression, including negative mood, fatigue, irritability, and low libido [156]. In addition, men treated with antiandrogen drugs have shown greater risk of developing MDD [157].

Interestingly, clinical studies have failed to show effectiveness for testosterone administration as an augmentation strategy in the management of depression in men [158], whereas administration of low-dose testosterone in women with treatment-resistant MDD has been observed to significantly improve depressive symptoms in comparison to placebo [159]. These paradoxical findings are consistent with the higher sensitivity of females to androgens, as higher androgen exposure has been noted to exert a definite negative influence in mood in females [160, 161]. Future studies should address these differences between gender and their clinical relevance.

## 4. Neurosteroid Pharmacotherapeutic Targets in Depression

Many neuron types—especially glutamatergic and GABAergic neurons—have shown *de novo* synthesis of steroidal messengers, termed neurosteroids, which have been observed to modulate neuronal excitability [162]. Allopregnanolone is the most well described of these molecules at present, which has been noted to have sedative and anesthetic effects as well as an impact in mood regulation [163]. As with all steroids, synthesis of APG requires the expression of several CYP enzymes in neurons [164].

APG appears to act as an allosteric modulator of the GABA-A receptor, as well as a negative feedback signal for the HPAA: In chronic stress conditions, lower APG levels have been linked with greater activation of the HPAA and a slower recovery towards homeostasis [165]. This in turn may be influenced by decreased basal activation of GABA-A receptors, a hypothesis consistent with the lower levels of APG found in the cerebrospinal fluid (CSF) and peripheral blood found in patients with various affective disorders, including MDD [166]. Selective serotonin reuptake inhibitors, tricyclic antidepressants, and mirtazapine have been observed to elevate APG levels, whereas electroconvulsive therapy, repeated transcranial magnetic stimulation, and sleep deprivation appear to have no such effect [167]. SAGE-217, a synthetic derivate of APG, is currently undergoing phase 2 clinical trials, with preliminary findings showing modest improvements in clinical scores for depression after short-term use [168]. Further studies are needed to explore the use of APG or its synthetic analogues in MDD.

The role of other neurosteroids in depression remains less clearly understood. Androstenedione may be particularly relevant as a regulator of the HPAA and androgen metabolism, but current findings are contradictory: Both direct and inverse relationships have been described between androstenedione and depressive symptoms [169, 170]. Future research may clear these discrepancies and present further potential therapeutic strategies involving neurosteroids.

## 5. Neuropeptide Pharmacotherapeutic Targets in Depression

### 5.1. Oxytocin and Arginine Vasopressin

**Figure 3:**
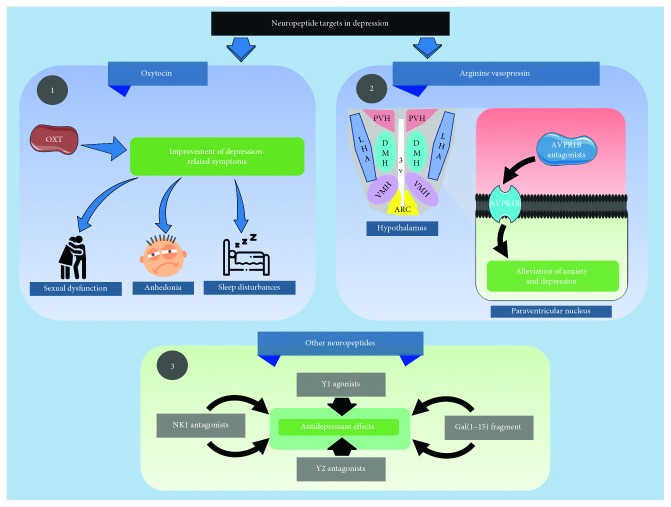
Neuropeptide pharmacotherapeutic targets in depression. OXT: oxytocin; LHA: lateral nucleus; PVH: paraventricular nucleus; DMH: dorsomedial nucleus; VMH: ventromedial nucleus; ARC: arcuate nucleus; AVPR1B: arginine vasopressin receptor 1B; NK1: neurokinin 1. Key findings regarding the current knowledge on neuropeptides in the neuropsychopharmacology of depression include the following: (1) Abundant preclinical and clinical evidence suggests oxytocin may significantly contribute to the improvement of depression-related symptoms such as sexual dysfunction, anhedonia, and sleep disturbances. (2) AVPR1B antagonists appear to reduce symptoms of anxiety and depression in both animal and human models. (3) Several modulators of neuropeptide signaling have shown antidepressant activity; however, further research is required to characterize their significance and utility.

In parallel to the study of neurosteroids, modern psychiatric research for future therapies has shown prominent interest in neuropeptides (Figure 3), especially oxytocin (OXT) and arginine vasopressin (AVP) [171]. Central oxytocin signaling exerts anxiolytic and antidepressant effects, whereas vasopressin tends to promote anxious and depressive behaviors. These opposing effects may underline the importance of the balanced activity of these neuropeptides regarding emotional regulation. Shifting this equilibrium towards oxytocin through positive social stimuli and psychopharmacotherapy may aid in the management of depression [172].

Indeed, in rodent studies, OXT has been clearly associated with positive social interaction [173, 174], and synthetic OXT has been shown to shift stress responses in rodents towards a more active coping style, after both central and peripheral administration [172]. Furthermore, OXT but not AVP, was recently shown to stimulate neuronal growth and to rescue glucocorticoid- or stress-induced suppression of neurogenesis in the hippocampus of adult rats [175]. In humans, OXT levels have been observed to be significantly lower in both psychotic and nonpsychotic depression [176], as well as bipolar depression [177]. Moreover, OXT appears to be particularly lower in subjects with the melancholic phenotype of depression, as ascertained in an mRNA expression study by Meynen et al. [178].

In addition, there is preclinical and clinical evidence that OXT may also contribute to the improvement of other depression-related symptoms, including sexual dysfunction: a study with ventral injections of OXT in male rats showed that stimulation of paraventricular DA receptors not only induces penile erection but also increases mesolimbic DA neurotransmission by activating oxytocin neurons. These findings suggest these mediators can powerfully influence both the consummatory and motivational/rewarding aspects of sexual behavior [179], anhedonia. [180], and possibly, sleep disturbances [181]. Furthermore, OXT but not AVP, was recently shown to stimulate neuronal growth and to rescue glucocorticoid- or stress-induced suppression of neurogenesis in the hippocampus of adult rats [175].

On the other hand, AVP appears to be an anxiogenic mediator [182]. AVP receptors have been long identified—AVPR1A, AVPR1B and AVPR2 [183]—though AVP can also bind to the structurally related OXT receptor (OXTR) with high affinity. AVR1A receptors are widely distributed on blood vessels and have also been found in the CNS, including the paraventricular nucleus, whereas AVPR2 receptors are predominantly located in the principal cells of the renal collecting system [172]. The AVP receptor family is G protein-coupled receptors: AVPR1A and AVPR1B are both coupled to Gq/11 and signal via phospholipase C [184, 185]. AVPR2 is coupled to Gs which, when activated, elevates cAMP levels by recruiting adenylate cyclase [186].

Studies on AVPR1B antagonists have yielded favorable results such as alleviation of anxiety and depression in animal and human models [187, 188]. In rat models, the AVP gene has been strongly correlated with trait anxiety [189]. Furthermore, clinical trials have associated the use of AVPR1B antagonists to HPAA modulation in subjects with MDD, along with amelioration of clinical symptoms [190].

### 5.2. Other Neuropeptides

Neurokinin 1 (NK1) antagonists were some of the earliest alternatives proposed for nonmonoamine-related biologic treatments for depression, following findings that linked chronic administration of MK-869, one of these molecules, with improvement of depressive symptoms [91, 191]. This led to the clinical study of aprepitant, another NK1 antagonist. Although initial reports were favorable, it failed to demonstrate efficacy in phase III clinical trials, discouraging further scientific interest in this matter [192].

However, more recent research suggests almost full central blockade of NK1 receptors is required for efficacy in the treatment of depression [193, 194]. Casopitant and orvepitant, two NK1 antagonists capable of much greater blockade have shown antidepressant efficacy in various isolated randomized trials [193–195]. This promising data have renewed interest in NK1 antagonists and other neuropeptide-related alternatives for depression.

Neuropeptide Y (NPY) is a very widespread neurotransmitter in the CNS, acting through a wide array of receptors [196–198]. In recent years, NPY has been reported to be decreased in depression, anxiety, and stress, in both plasma [199] and CSF [200]. Conversely, antidepressant treatment has been linked with increased NPY levels [201].

NPY-related therapeutic interventions have gained attention I light of these findings. Data from mice models are abundant: central administration of NPY has been associated with reduced immobility and longer swimming times in forced swim tests [202], along with other similar correlates [203–205], while Y1 receptor knockout mice tend to show opposite results [206]. In contrast, Y2 and Y4 receptor knockout mice have shown more resilient phenotypes in these tests [206, 207], and the infusion of Y2 antagonists—as well as Y1 agonists—has been related with antidepressant effects [202]. This would suggest a differential role for distinct NPY receptor types, a promising hypothesis for future research.

Galanin has also been proposed to intervene in the neurobiology of depression [208, 209]. The galanin system includes three major G protein-coupled receptors (GALR1, GALR2, and GALR3), all which are widespread in the CNS and tend to colocalize with monoamine receptors, forming heteroreceptor complexes [210]. Thus, galanin signaling is an important modulator of neurotransmission. Galanin overexpression has been described in depression and stress [211], and serum galanin levels have been suggested as a biomarker for depression [212]. In a siRNA GALR1 and GALR2 knockdown rat model, coadministration of fluoxetine with the Gal (1–16) fragment obtained greater antidepressant effects [213]. Several other studies have found similar results with various galanin ligands [214–217]. Nevertheless, understanding of the neurobiology of galanin is still incipient, in particular regarding mood regulation.

## 6. Pharmacotherapeutic Targets for Depression in Reward Neurocircuits

The reward system encompasses various neurocircuits which mediate motivational behavior and learning in response to external and internal stimuli [218]. Anatomically, this system originates in the ventral tegmental area (VTA) and projects to the nucleus accumbens (NAcc), lateral hypothalamus, lateral septum, hippocampus, amygdala, PFC, and anterior cingulate cortex (ACC) [219]. Preclinical and clinical findings in neuroimaging have demonstrated that the anhedonia and loss of motivation found in depression is closely linked with decreased size and functionality of several of the nuclei in the reward system, in particular the NAcc and ACC [220, 221], along with reduced dopaminergic neurotransmission, one of the pillars of the monoamine hypothesis [222, 223].

**Figure 4:**
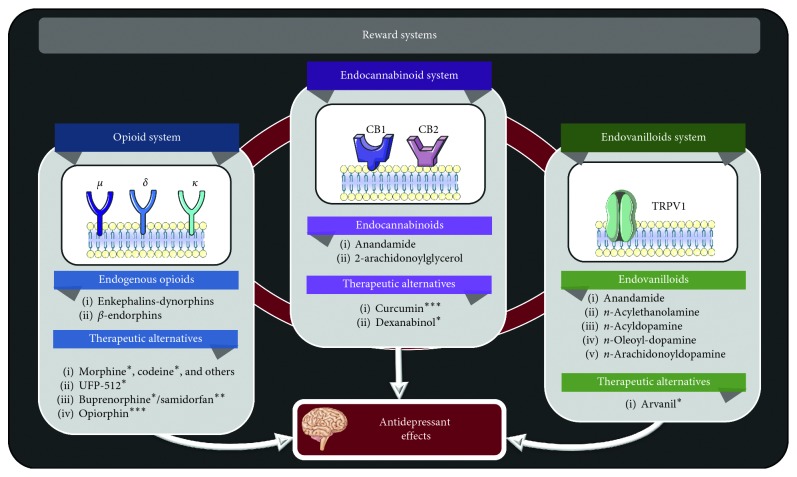
Pharmacotherapeutic targets for depression in reward neurocircuits. ^∗^Agonist. ^∗^^∗^Antagonist. ^∗^^∗^^∗^Modulator. *μ*: *µ*-opioid receptor. *δ*: *δ*-opioid receptor. κ: κ-opioid receptor. CB1: cannabinoid receptor 1. CB2: cannabinoid receptor 2. TRPV1: transient receptor potential cation channel V1. Research on pharmacotherapeutic targets for depression in the reward system remains principally preclinical. Currently available results presume some potential clinical utility for these substances for the treatment of depression, with varying degrees of efficacy and differing pharmacological profiles.

Functionally, reward processing involves two interrelated components: motivational processing, which centers attention and behavior on rewarding stimuli and fundamentally involves dopaminergic neurotransmission; and hedonic processing, which mediates the pleasurable reaction to these stimuli, and involves GABAergic, opioid, endocannabinoid (EC), and endovanilloid (EV) signaling throughout the NAcc, ventral pallidum, insular cortex, and orbitofrontal cortex [224, 225]. The crosstalk among these systems is complex and remains to be fully elucidated [226, 227]. However, specific components in these circuits have already emerged as potential targets in the neuropsychopharmacological approach to depression (Figure 4).

Dysregulation of opioid signaling has long been associated with depression, as indeed, opioids were historically trialed in the management of this disorder long before the introduction of modern AD [228–230]. Opioid receptors are in the G protein-coupled receptor superfamily, including three distinct types—mu (*µ*), delta (*δ*), and kappa (κ)—with different physiologic roles. Endogenous opioids are their native ligands, a group of peptides characterized by sharing a specific NH-terminal sequence (Tyr-Gly-Gly-Phe). Endogenous opioids have been classified as enkephalins, dynorphins, and *β*-endorphin according to their structure and their affinity for different receptor types [231].

Opioid peptides and their receptors are amply distributed in the central and peripheral nervous systems, intervening in nociception, analgesia, endocrine, and immunologic regulation, mood regulation, as well as hedonic and motivational processing, and modulation of addictive behavior [229]. In the CNS, opioid receptors are predominantly expressed in the brainstem, limbic nuclei, and cortex; the latter two being particularly relevant to mood disorders [232]. Indeed, neuroimaging and pathology studies—both in living subjects and *postmortem*—in subjects with depression and suicidal behavior, have shown structural and functional alterations in opioid signaling, especially in the PFC, NAcc, and ACC [233–237].

Although both misuse and chronic, high-dose treatment with opioids have been consistently linked increased incidence, relapse, and recurrence of depression [238–241], more nuanced used may be beneficial in this regard. In preclinical research, various opioid agonists such as morphine, codeine, levorphanol, methadone, and tramadol have been observed to yield better outcomes than commonly available antidepressants in mice models evaluating depressive behavior, with these effects being reversible by naloxone, an opioid antagonist [242]. The antidepressant efficacy of opioids may depend specifically on δ-receptor agonism, as observed in mice and rats treated with intracerebroventricular and intraperitoneal UFP-512, a *δ*-selective opioid agonist [243]. Because of the tolerance, dependence and abuse potential associated with unopposed and selective *δ* agonism [244], interest for the use of opioids in this situation has shifted to combined opioid agonists/antagonists. In particular, the coadministration of buprenorphine, a *δ*-receptor partial agonist and antagonist of *δ* and κ receptors, along with samidorfan, a *δ*-selective antagonist, has been associated with rapid antidepressant effects [245].

Another opioid-related potential pharmacological target for depression has been found in opiorphin, an inhibitor of Zn-ectopeptidase, neutral endopeptidase, and aminopeptidase N, all of which intervene in the rapid inactivation of enkephalins [246]. In preclinical models, opioid administration has been linked with attenuated depressive responses, with this effect being reversible by the administration of naldrindole, a *δ*-selective antagonist [247], or naloxone [248]. Despite these promising results, research in humans remains scarce, and further study is required to assuage concerns related to misuse of these substances.

The endocannabinoid system also has an important role in mood regulation, and levels of EC metabolites in CSF have been correlated with severity of depression [237]. EC are lipophilic substances synthesized on demand in various nuclei within the reward circuits, the principal molecules being anandamide, which also shows affinity for vanilloid receptors, and 2-arachidonoylglycerol [249, 250]. Cannabinoid receptor 1 is localized in glutamatergic and GABAergic synapses throughout basal nuclei, while cannabinoid receptor 2 can be found both in the CNS and in immune cells, posing a provocative link between depression and systemic health [251]. Polymorphisms in the genes encoding these receptors have been correlated with specific treatment resistance [252], severe depression symptoms, [253] anxiety, and suicidal behavior [254]. Furthermore, levels of cannabinoid receptor ligands in CSF have been determined to be increased in subjects with depression after undergoing electroconvulsive therapy [255].

Although clinical research remains scarce regarding EC modulation in depression, preclinical findings appear promising. Recently, Xiaolie et al. investigated the role of curcumin, a cannabinoid receptor modulator, and dexanabinol-loaded solid lipid nanoparticles in depression treatment in mice and cultured cells. Their findings showed this compound increased EC expression and potentiated cannabinoid receptor activation, with increased activity in the ERK1/2 pathway, a key cascade in the activity of many AD [256].

Finally, endovanilloids are endogenous substances which possess a vanillyl group and show affinity for the TRPV1 receptor, the main molecules in this category being anandamide, *n-*acylethanolamines, *n*-acyldopamines, *n*-oleoyl-dopamine, and *n*-arachidonoyl-dopamine, as well as some lipoxygenase derivatives of arachidonic acid like 12-hydroperoxyeicosatetraenoic acid [257]. Though their physiology remains obscure, endovanilloids have been implicated in locomotion, pain modulation, and regulation of emotion, cognition, and behavior; pharmacological modulation of TRPV1 receptors has been speculated to be useful in the treatment of pain, anxiety, depression, and various neurological disorders [258]. TRPV1 receptors have been shown to modulate input to the locus coeruleus, a key area implicated in the regulation of mood, the stress response, and memory processing [259].

In preclinical studies, TRPV1 knockout mice tend to exhibit reduced immobility time and reduced latency times in the novelty-suppressed feeding paradigm, consistent with a decreased depressive response [260]. TRPV1 receptor modulation has been observed to boost the effect of AD [261], and arvanil, a synthetic agonist ofTRPV1 and cannabinoidreceptors, appears to induce significant antidepressant effects in mice [262]. Future research in humans in this field should clear the significance and utility of the neuropsychopharmacological modulation of the reward system in depression.

## 7. Conclusions

Expanding comprehension of depression as a neuroendocrine disorder has injected much-needed hope into the landscape of neuropsychopharmacology. In particular, glutamate-based alternatives may be the most feasible in the near future, with promising and active clinical trials at the moment evaluating the use of both intravenous and oral ketamine, along with several other related molecules [263, 264]. Likewise, nonpharmacological interventions, both well-established—including lifestyle modifications and electroconvulsive therapy—and more novel, such as deep brain stimulation, transcranial magnetic stimulation, and psychosurgery, should not be discounted, as their place in the treatment of depression has become better characterized in recent years.

Furthermore, evolving views on the pathophysiology of depression suggest pharmacological and nonpharmacological interventions centered on the immunologic, metabolic, and cardiovascular aspects of this disorder may break new ground in the field of psychiatric therapeutics in the future. Therefore, although present therapeutic outcomes require urgent improvement, there may be enough forthcoming innovation to remain optimistic regarding the conundrum of treatment alternatives in depression.
